# Inclusion of patient care technicians in hemodialysis patient care conferences: a pilot implementation study

**DOI:** 10.1186/s12882-026-05087-6

**Published:** 2026-05-30

**Authors:** Laura C. Plantinga, Jennifer Evans, Susan Chapman, Clarica Douglas-Ajayi, Danilo Concepcion, Bernard G. Jaar, Delphine Tuot

**Affiliations:** 1 Department of Medicine, University of California San Francisco, 2540 23rd Street, Pride Hall #4403, San Francisco, CA 94110 USA; 2 Department of Social and Behavioral Sciences, University of California San Francisco, San Francisco, CA USA; 3 National Association of Nephrology Technicians/Technologists, Dayton, OH USA; 4 Independent Dialysis Consultant, Chino Hills, CA USA; 5 Department of Medicine, Johns Hopkins University, Baltimore, MD USA

**Keywords:** Dialysis, Workforce, Patient care technician, Interdisciplinary care, Pilot, Implementation

## Abstract

**Background:**

Including patient care technicians (PCTs), who spend the most chairside time with patients receiving hemodialysis, in monthly interdisciplinary patient care conferences (PCCs) may provide the dialysis care team with valuable patient information and improve PCTs’ sense of belonging on the team. We assessed the implementation of including PCTs in PCCs (“In-Tech”).

**Methods:**

In this pre-post pilot study, In-Tech was implemented at both clinics for 6 months (March–August 2025) by dialysis care team members (nephrologists, nurses, social workers, dietitians, PCTs) at two independent dialysis clinics. In pre- and post-intervention surveys of PCTs and other dialysis care team members, organizational readiness for, acceptability of, appropriateness of, and feasibility of In-Tech were measured via items from validated instruments, and the value of In-Tech was measured via ad hoc items.

**Results:**

Of 21/37 (pre-/post-implementation) survey respondents, 33%/32% were > 50 years old, 57%/64% were women, 35%/41% were Asian, and 25%/35% were Pacific Islander. Most (> 80%) found In-Tech to be acceptable; organizational readiness (76–81% vs. 65–73%), appropriateness (81% vs.73%), and feasibility (81% vs. 70%) were higher in pre- vs. post-intervention surveys. PCTs were less likely than other care team members to express the value of PCTs attending PCCs (73–80% vs. 86–96%) and to report they were encouraged to share their thoughts (60% vs. 100%) or that their input was valued (60% vs. 100%) during PCCs. Open-ended responses emphasized the value of the patient–PCT relationship, difficulty scheduling PCCs to accommodate PCTs, the need for PCT guidance in their contributions to PCCs, and the desire to continue In-Tech beyond the pilot.

**Conclusions:**

While including PCTs in PCCs appears to be implementable and valuable to the care team, future studies should address implementation challenges and ensure that PCT input is explicitly valued by the care team.

**Clinical trial number:**

Not applicable.

**Supplementary Information:**

The online version contains supplementary material available at 10.1186/s12882-026-05087-6.

## Introduction

Dialysis patient care technicians (PCTs) are frontline care providers for the nearly 500,000 U.S. patients receiving in-center hemodialysis (HD) [1], and, under the supervision of registered nurses (RNs), they are responsible for several critical aspects of HD care, including cannulation of the vascular access, operation of complex dialysis machines, and monitoring of vital signs throughout the procedure. In our recent national survey of dialysis PCTs, more than half (58%) reported burnout, and only 47% intended to continue working as PCTs in 3 years [2]. Perceptions of inadequate supervisor support and lack of respect from colleagues were cited as top contributing factors to burnout and turnover intention [2]. Better integration of the entire Centers for Medicare & Medicaid Services (CMS)-mandated [3] interdisciplinary dialysis care team (including RNs and PCTs, but also nephrologists, social workers, and dietitians) could help mitigate these negative perceptions and attract and retain a stable workforce of dialysis PCTs.

One approach to improving dialysis care team integration would be to include PCTs in monthly interdisciplinary patient care conferences (PCCs), during which individual cases are discussed by the care team. This inclusion could help demonstrate the value of PCTs’ contributions to HD care to the care team and PCTs themselves. Furthermore, given the substantial time PCTs spend with patients on HD (up to 4 h three times a week), they may have timely and detailed information about patients’ clinical and social needs, which, if shared with the care team, could improve the patient-centeredness and delivery of HD care.

We hypothesized that the inclusion of PCTs in monthly interdisciplinary PCCs would improve PCTs’ perceptions of collegial respect and support, as well as facilitate an exchange of information that would optimize short-term patient outcomes (e.g., patient adherence to dialysis, use of permanent vascular access) that are associated with reduced HD mortality and morbidity [4–8]. However, given that such an intervention requires changes in established workflows, our first step was to assess whether it can be implemented successfully. Here, we report on the implementation of a 6-month pragmatic pilot [*In*clusion of Dialysis Patient Care *Tech*nicians in Interdisciplinary Care Conferences for Patients Receiving Hemodialysis (“In-Tech”)] at two dialysis clinics.

## Methods

### Study population, recruitment, and data sources

Two independent dialysis clinics (Clinic A: 14 HD stations; Clinic B: 13 HD stations; no shared personnel) in San Francisco, California, recruited by the principal investigator (L.P.), agreed to implement In-Tech. Characteristics of the clinics are listed in Supplementary Table 1 (see Additional File 1). Study data pertinent to organizational readiness, feasibility, and acceptability were collected from pre- and post-intervention surveys of PCTs and other dialysis care team members (nephrologists, nurse managers, RNs, social workers, and dietitians), which were developed for this study and included items adapted from validated surveys and ad hoc items; survey items, their sources, and their modifications (as applicable) are listed in Additional File 2. At both time points, individual survey participants were recruited via email invitation, in person, or through flyers displayed in the dialysis clinics, and they were incentivized with $25 gift cards for completed surveys. Given the sensitive nature of some of the data collected, survey participants were not tracked longitudinally. Additional data on PCT attendance at PCCs and the number of patient cases per PCC for which they were present were collected monthly from PCT champions (PCT who was tasked with encouraging their peers to attend PCCs) and/or nurse managers at the clinics throughout the pilot and were used for both intervention fidelity and reimbursement tracking purposes.

### Intervention

In-Tech was implemented by the dialysis care teams over 6 months (March 2025–August 2025). The research team introduced In-Tech at both clinics during team huddles prior to the pilot, to familiarize the staff with the research team and to encourage initial buy-in for In-Tech. Also prior to the start of the pilot, care teams identified PCT study champions at each clinic. Prior to the start of the pilot, these PCT champions, along with the nurse managers, met with our research team to gain a better understanding of the project and its purpose; PCTs were further encouraged to meet with our PCT consultants (C.D-A. and D.C.) to strategize methods for maximizing PCT participation at their clinic. All PCTs were additionally encouraged to attend PCCs via flyers posted in the dialysis clinics and incentives (one $25 gift card for each individual patient case at which they were present during that month’s PCCs).

### Outcomes

Our primary outcomes were related to the implementation potential of In-Tech and were assessed via pre- and post-implementation surveys of PCTs and other dialysis care team members. These outcomes were guided by the integrated Reach, Effectiveness, Adoption, Implementation and Maintenance (RE-AIM) [9] and Practical, Robust Implementation and Sustainability Model (PRISM) [9] frameworks, which assess implementation and the contextual factors affecting implementation, respectively.

#### RE-AIM implementation outcomes

##### Reach

Survey items assessing the reach of In-Tech included: whether participants had heard of In-Tech (pre- and post-implementation surveys), and whether participants had attended PCCs (PCTs) or attended PCCs at which PCTs were present (other staff; post-implementation survey).

##### Effectiveness

Ad hoc structured and open-ended items were included in the post-implementation survey to assess the perceived value of In-Tech (Supplementary Table 2; see Additional File 1). Items regarding perceptions of the PCCs were assessed only among those who reported attending at least one PCC (PCTs) or one PCC with a PCT present (other staff) during the pilot.

##### Adoption

Adoption of In-Tech was assessed by identifying which PCTs participated in PCCs and which dialysis care team members responded to the surveys.

##### Implementation

Implementation was assessed with measures of feasibility and fidelity. For feasibility, two adapted items from the Feasibility of Implementation Measure (FIM),^10^ as well as one ad hoc item addressing the potential disruption of patient care by In-Tech, were included in both the pre- and post-intervention surveys (Supplementary Table 2; see Additional File 1). Fidelity to the intervention was assessed using data collected monthly from nurse managers and/or PCT champions and was operationalized as the percentage of PCTs at the clinic who attended PCCs each month, the percentage of PCCs in the month with at least one PCT present, and the percentage of patient cases at which PCTs were present each month.

##### Maintenance

The potential for maintenance, or sustainability, was assessed as the acceptability of the intervention. Two adapted items from the Acceptability of Intervention Measure (AIM) [10] were included in both the pre- and post-intervention surveys, with wording adapted to reflect the timing of the survey (Supplementary Table 2; see Additional File 1). Responses to open-ended items that mentioned continuing In-Tech beyond the pilot were also used to assess the potential for maintenance.

#### PRISM contextual factors

##### Organizational readiness and appropriateness

Organizational readiness was assessed using adapted items from the Organizational Readiness for Implementing Change (ORIC), which was designed for healthcare settings and includes measures of commitment to the change and the value placed on the change [11]. Items were included in both the pre- and post-implementation surveys and reworded as needed to reflect the timing of the survey (pre- or post-intervention; Supplementary Table 2; see Additional File 1). Additionally, staff perceptions of the appropriateness of In-Tech for their clinic were assessed using two adapted items from the Intervention Appropriateness Measure (IAM) [10] in both the pre- and post-intervention surveys (Supplementary Table 2; see Additional File 1).

##### Other variables

Other contextual variables were collected about the clinics and survey participants. Clinic characteristics, such as number of shifts and quality measure performance, were obtained directly from nurse managers and/or from publicly available information via the CMS. The demographics of survey participants (age group, sex, race, ethnicity, country of birth, and language spoken at home) and work characteristics (clinic role, time in role, patient caseload, hours worked at clinic) were self-reported in the pre- and post-implementation surveys. We also collected a variety of work experience data, including: overall job satisfaction, burnout [12], turnover intention, intrinsic and extrinsic rewards [13, 14], respect/autonomy [13], experiences of discrimination and violence at work [15], and perceptions of patient care [13].

### Statistical analysis

Survey responses were tabulated and summarized as appropriate, both overall and by pilot clinic. Responses to structured feasibility items were dichotomized as agree/somewhat agree, completely agree/agree, or strongly agree/agree vs. all other responses, as appropriate, and percentages with “agree” responses were tabulated. Themes in open-ended items were quantified as a percentage of mentions per item (Supplementary Table 2; see Additional File 1). Analyses were performed using Stata v. 19.5 (College Station, TX).

## Results

### Characteristics and work experiences reported by survey participants

Of the 21 pre-implementation survey respondents [21/50 (42.0%) overall response rate; 11/23 (47.8%) and 10/27 (37.0%) in Clinics A and B], about half (52.4%) were aged 35–49 years, 57.1% were women, and 35.0% and 25.0% were Asian and Hawaiian/Pacific Islander race, respectively (Table 1). Similarly, of the 37 post-implementation survey respondents [37/49 (75.5%) overall response rate; 13/23 (56.5%) and 24/26 (92.3%) in Clinics A and B], 51.4% were aged 35–49 years, 63.9% were women, and 41.7% and 36.1% were of Asian and Hawaiian/Pacific Islander race. Most respondents on pre- and post-implementation surveys were PCTs (28.6% and 40.5%) and nurses (RNs, 19.0% and 21.6%; and charge nurses, 9.5% and 5.4%; Table 1). Characteristics were similar across clinics for each survey (Supplementary Table 3; see Additional File 1).

**Table 1:** Characteristics of survey participants

Participant characteristic	Survey	
	Pre-Intervention (*N* = 21)	Post-Intervention (*N* = 37)
**Demographic**		
Age, *n* (%)		
18–34 years	3 (14.3%)	5 (13.5%)
35–49 years	11 (52.4%)	19 (51.4%)
≥50–64 years	7 (33.3%)	13 (35.1%)
Sex, *n* (%)		
Women	12 (57.1%)	23 (63.9%)
Men	9 (42.9%)	13 (36.1%)
Race^a^		
American Indian/Alaskan Native	2 (10.0%)	1 (2.8%)
Asian	7 (35.0%)	15 (41.7%)
Black	2 (10.0%)	3 (8.3%)
Hawaiian/Pacific Islander	5 (25.0%)	13 (36.1%)
White	4 (20.0%)	4 (11.1%)
Ethnicity, *n* (%)		
Hispanic	2 (10.5%)	4 (12.5%)
Non-Hispanic	17 (89.5%)	28 (87.5%)
Other language spoken at home, *n* (%)		
Yes	10 (47.6%)	23 (63.9%)
No	11 (52.4%)	13 (36.1%)
**Work-related**		
Role,^a^ *n* (%)		
Medical director	0 (0.0%)	1 (2.7%)
Nephrologist	0 (0.0%)	2 (5.4%)
Nurse manager	2 (9.5%)	0 (0.0%)
Charge nurse	2 (9.5%)	2 (5.4%)
Registered nurse	4 (19.0%)	8 (21.6%)
Social worker	2 (9.5%)	3 (8.1%)
Dietitian	3 (14.3%)	3 (8.1%)
Patient care technician	6 (28.6%)	15 (40.5%)
Other	3 (14.3%)	5 (13.5%)
Time in role, *n* (%)		
<1 year	3 (14.3%)	3 (12.1%)
1–5 years	7 (33.3%)	13 (35.1%)
>5 years	11 (52.4%)	21 (56.8%)
Working at multiple clinics, *n* (%)		
Yes	4 (19.0%)	12 (32.4%)
No	17 (81.0%)	25 (67.6%)
Hours worked at clinic, *n* (%)		
≤40 h/week	16 (76.2%)	28 (75.7%)
>40 h/week	5 (23.8%)	6 (16.2%)
Median (IQR) patient caseload	20 (15, 56)	30 (12, 58)

IQR, interquartile range (25th-75th percentile)

^a^Totals may add to > 100% since individuals could select more than one role

Participants reported high overall job satisfaction (median rating = 8 on both pre- and post-implementation surveys), despite a high prevalence of reported burnout (66.7% and 50.0%) and turnover intention within the next year (23.9% and 18.9%; Table 2). About three-quarters (71.4% and 73.0%) agreed that pay was good (extrinsic reward), and 85.7% and 83.8% agreed their patients gave them a reason to come to work every day (intrinsic reward). Most agreed that their coworkers treated them as an equal member of the healthcare team (respect; 90.5% and 88.9%) and that it was their own responsibility to decide how their job gets done (autonomy; 81.0% and 78.4%). Nearly half reported having experienced discrimination at work (47.6% and 43.2%). More participants in the pre- vs. post-intervention survey perceived patient care at the clinic as good (e.g., 90.5% vs. 78.4% agreed patient areas were kept clean; Table 2). While participants from Clinic B were less likely than those in Clinic A to agree that pay was good, they were more likely to report better career mobility, respect, and autonomy, and they were less likely to agree they had experienced discrimination (Supplementary Table 4; see Additional File 1).

**Table 2:** Perceptions and experiences of work among survey participants

Perceptions/experiences	Survey	
	Pre-Intervention (*N* = 21)	Post-Intervention (*N* = 37)
Job satisfaction rating (0–10, 10 = highest satisfaction), median (IQR)		
Overall job satisfaction	8 (7, 9)	8 (7, 9.5)
Burnout, *n* (%) monthly or more frequently		
Experiencing burnout	14 (66.7%)	18 (50.0%)
Turnover intention, *n* (%) no/not sure		
Plan to be in the same job in 1 year	5 (23.8%)	7 (18.9%)
Plan to be in the same clinic in 1 year	6 (28.6%)	6 (16.2%)
Extrinsic value, *n* (%) strongly agree/agree		
The pay is good	15 (71.4%)	27 (73.0%)
The benefits are good	19 (90.5%)	30 (83.3%)
Support for continuing education is good	15 (71.4%)	27 (73.0%)
Job security is good	16 (76.2%)	29 (78.4%)
Intrinsic value, *n* (%) strongly agree/agree		
My patients give me a reason to come to work every day	18 (85.7%)	31(83.8%)
Expectations and mobility, *n* (%) strongly agree/agree		
Chances for promotion are good	9 (42.9%)	21 (56.8%)
Promotions are handled fairly	8 (38.1%)	23 (62.2%)
My job measures up to the sort of job I wanted when I took it	16 (76.2%)	28 (75.7%)
Respect, *n* (%) strongly agree/agree		
My supervisor treats me as an equal member of the healthcare team	16 (76.2%)	30 (81.1%)
My supervisor listens carefully to my observations and opinions	17 (81.0%)	28 (77.8%)
My other coworkers treat me as an equal member of the healthcare team	19 (90.5%)	32 (88.9%)
My other coworkers listen carefully to my observations and opinions	20 (95.2%)	32 (86.5%)
Autonomy, *n* (%) strongly agree/agree		
It is basically my own responsibility to decide how my job gets done	17 (81.0%)	29 (78.4%)
I have input into patient care planning	16 (76.2%)	29 (78.4%)
Discrimination/violence, *n* (%) rarely/sometimes/often/always		
I have been treated unfairly at work because of my race, ethnic group, gender, age, disability status, or other personal characteristic	10 (47.6%)	16 (43.2%)
I have experienced threats of violence or physical abuse, or actual abuse, at work	8 (38.1%)	11 (29.7%)
Perceptions of patient care, *n* (%) strongly agree/agree		
Sometimes our staff take out their bad days on the patients	1 (4.8%)	3 (8.1%)
Some staff are hostile toward patients	6 (30.0%)	5 (14.3%)
I treat patients like I would like to be treated	20 (95.2%)	33 (91.7%)
Patient areas are kept clean here	19 (90.5%)	29 (78.4%)
All personnel take responsibility for answering patient alarms	17 (81.0%)	27 (73.0%)

IQR, interquartile range (25th-75th percentile)

### Implementation of In-Tech

#### RE-AIM implementation outcomes

*Reach of In-Tech*. Prior to the pilot, 57.1% of survey participants overall reported having heard about In-Tech (90.9% for Clinic A, 20.0% for Clinic B); post-implementation, 83.8% reported having heard of it (92.3% for Clinic A, 79.2% for Clinic B). Most survey participants reported participating in at least one PCC (PCTs: 10/15, 66.7%) or participating in a PCC where at least one PCT was present (other staff: 15/20, 75.0%).

**Fig. 1:**
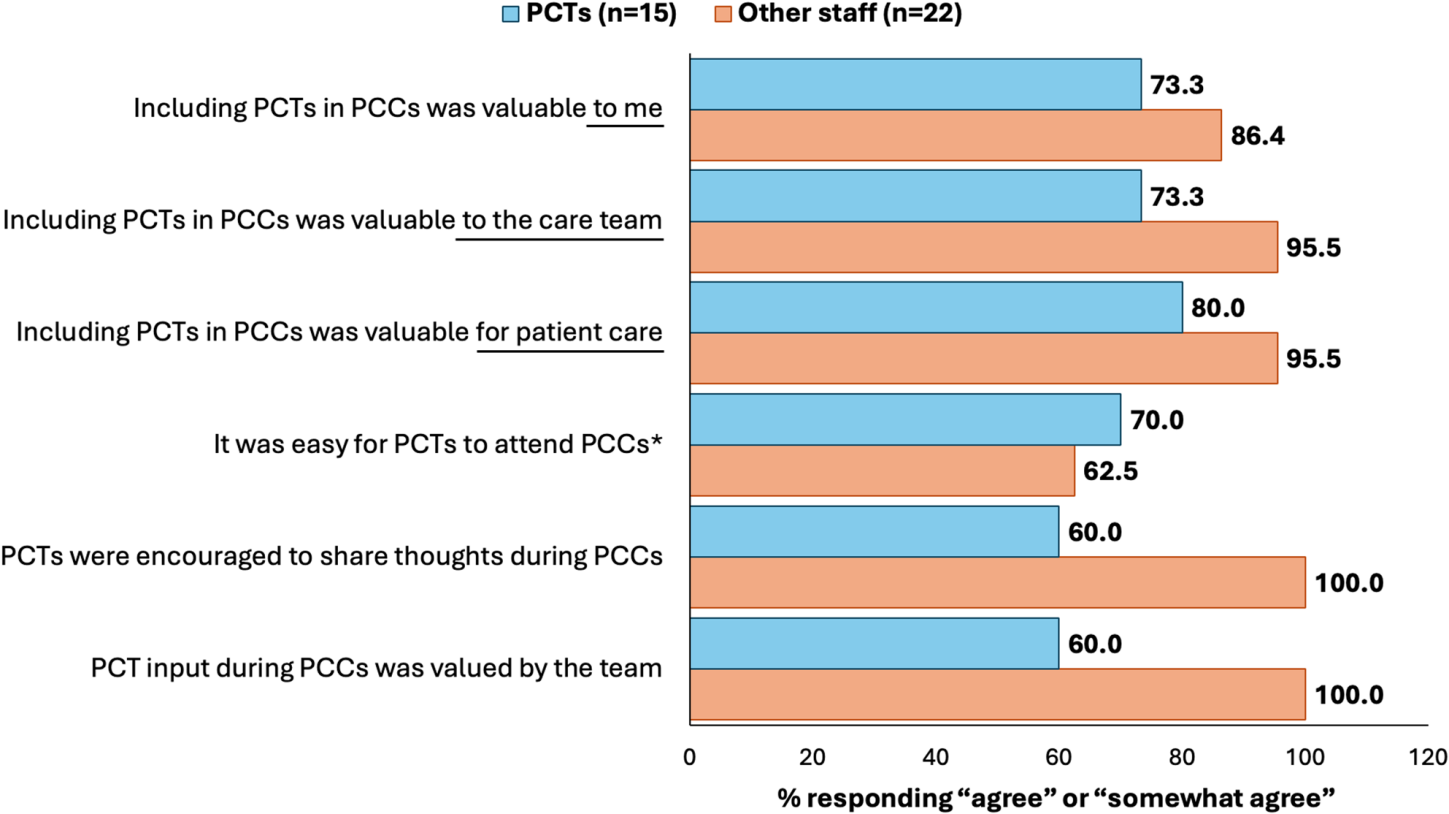
Post-implementation perceptions of In-Tech among survey respondents *Asked of nurses/nurse managers only

*Effectiveness*. Among PCTs, 73.3%, 73.3%, and 80.0% reported that In-Tech was valuable to them, to the care team, and to patient care, respectively; these percentages were higher among other staff (86.4%, 95.5%, and 95.5%; Fig. 1). While PCTs were more likely than other staff to report it was easy for them to attend conferences (70.0% vs. 62.5%), they were less likely to report that PCTs were encouraged to share their thoughts during PCCs (60.0% vs. 100%) and that PCT input at PCCs was valued (60.0% vs. 100%; Fig. 1). In open-ended responses regarding the importance of In-Tech, 100% agreed that inclusion of PCTs in PCCs was important, with 42.9% emphasizing the importance of the PCT-patient relationship, allowing for sharing of information that is often not documented; 42.9% reporting that PCTs are part of the care team; 17.9% specifically mentioning the sharing of information about fistulae; and 10.7% valuing the different perspective offered by PCTs (Supplementary Fig. 1A; see Additional File 1).

*Adoption.* At Clinic A, 1/14 PCTs (7.1%) attended a PCC in the first month, after which no Clinic A PCTs attended any PCCs; while at Clinic B, 14.3–30.8% of PCTs attended PCCs throughout the pilot *(*Supplementary Table 5; see Additional File 1). All of the scheduled PCCs at Clinic B were attended by at least one PCT, with 58.3–100% of patient cases having at least one PCT present. While PCTs, RNs, social workers, and dietitians participated in surveys at both time points, nephrologists and medical directors only participated in the post-implementation survey (Table 1), after direct recruitment by the PI (L.P.); and these roles were only represented in Clinic B (Supplementary Table 3; see Additional File 1).

**Fig. 2:**
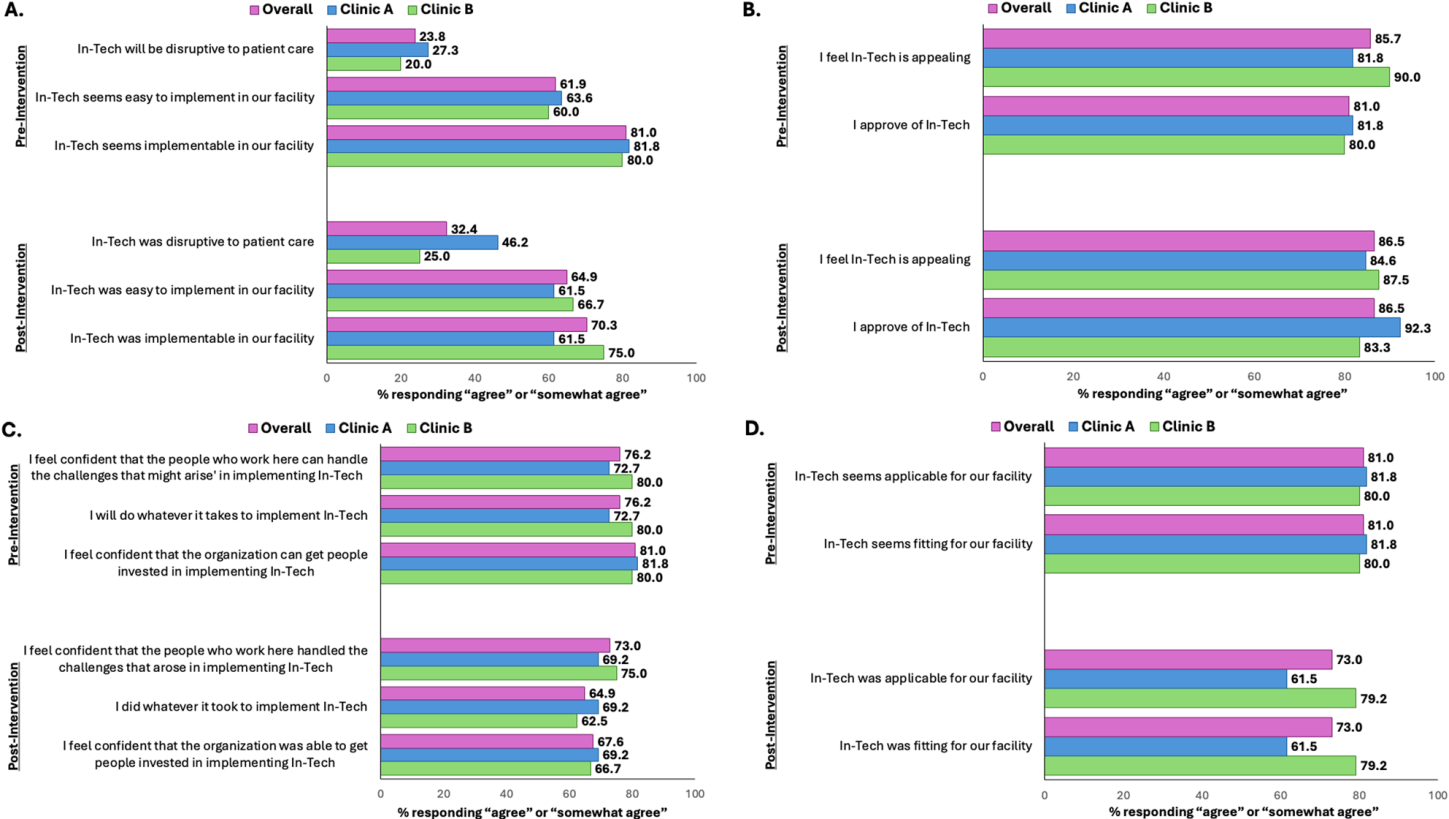
Implementation outcomes, including feasibility of In-Tech (**A**), acceptability of In-Tech (**B**), organizational readiness for In-Tech (**C**), and appropriateness of In-Tech (**D**), among survey respondents, pre- and post-intervention

*Implementation*. Most participants agreed that In-Tech was implementable in their facility in the pre- and post-intervention surveys (81.0% vs. 70.3%); fewer agreed that it was easily implementable (61.9% and 64.9%; Fig. 2A). About one-quarter agreed that In-Tech would be disruptive to patient care (23.8%) at the start of the pilot, while 32.4% agreed it was disruptive at the end of the pilot.

*Maintenance.* Most participants in the pre- and post-intervention surveys reported that In-Tech was appealing (85.7% and 86.5%) and that they approved of In-Tech (81.0% and 86.5%), and patterns were similar across clinic (Fig. 2B). In open-ended responses, 71.4% reported that In-Tech should continue (Supplementary Fig. 1B; see Additional File 1).

#### PRISM contextual factors

Organizational readiness was high prior to the start of the pilot (Fig. 2C), with 76.2–91.0% reporting that they agreed the clinic could implement In-Tech. After the pilot, these percentages were lower (64.9–73.0%). While scores for Clinic A were slightly lower than those for Clinic B, the pre- and post-implementation patterns were similar for both. More participants in the pre- vs. post-intervention surveys reported that In-Tech was applicable to their facility (81.0% vs. 73.0%) and that it was fitting for their facility (81.0% vs. 73.0%; Fig. 2D). While percentages were similar across clinics in the pre-intervention survey, participants in Clinic A were less likely than those in Clinic B to agree with these statements.

In open-ended responses, scheduling was the most frequently reported challenge (87.0%); understaffing/PCT coverage (30.4%), the lack of inclusion of PCTs in other clinic meetings (8.7%), and lack of clarity about the role of the PCT in PCCs (4.3%) were also reported (Supplementary Fig. 1C; see Additional File 1). Suggested improvements included better scheduling/coverage (33.3%), a communication guide or template for PCTs to use during PCCs (25.0%), and involvement of PCTs in other meetings and aspects of care (16.7%) (Supplementary Fig. 1B; see Additional File 1).

## Discussion

In this 6-month, two-clinic pilot to assess the implementation of In-Tech, we found that most pre-pilot participants (81%) considered In-Tech to be implementable, while 62% felt it would be easily implementable. Only 24% felt it would be disruptive to patient care. After the pilot, fewer reported it was implementable (70%) and more reported it was disruptive to patient care (32%). Similarly, more pre- vs. post-implementation survey participants indicated that the clinics were ready for In-Tech (76–81% vs. 65–73%) and that In-Tech was appropriate for their clinic (81% vs. 73%). However, most (> 80%) dialysis staff across clinics found In-Tech to be acceptable before and after the pilot. Furthermore, following implementation, most PCTs and other dialysis care team members reported that In-Tech was valuable to them personally (73% and 86%), to their team (73% and 96%), and to patient care (80% and 96%). The desire to continue In-Tech beyond the pilot was also expressed in open-ended items.

Overall, our results suggest that dialysis care team members recognize the value in including PCTs in PCCs. However, implementation challenges should be addressed in future studies to assess the effectiveness of this or similar interventions. In open-ended item responses, the most commonly reported challenge to implementing In-Tech was difficulty scheduling PCCs during times that would allow PCTs to attend; the next most commonly reported challenge was inadequate PCT staffing and coverage. Although the CMS Conditions for Coverage for ESRD Facilities mandate “an adequate number of qualified personnel are present whenever patients are undergoing dialysis so that the patient/staff ratio is appropriate to the level of dialysis care given and meets the needs of patients,” [3] to our knowledge, no federal or state policies specifically mandate a maximum patient-to-PCT ratio. In fact, proposed California legislation to limit the number of patients per PCT per shift to three was withdrawn due to lack of support [16]. In the absence of PCT staffing mandates and with increasing demand for (but rising turnover among) PCTs, the number of open PCT positions has been steadily increasing [1, 17]. Additionally, the structure of the typical PCT workday, including starting and stopping treatments (with no minimum time mandated between patients [16]) and continuous monitoring of patients who are dialyzing on staggered schedules, can leave little time for activities outside of direct patient care. Thus, many clinics may often be in the position of not being able to “spare” a PCT who is on the floor monitoring patients. While shift changes might be ideal times for scheduling on-site PCCs, the limited availability of nephrologists (who are often not on site except for physician rounds and PCCs) likely also limits scheduling options for these physician-led conferences; virtual PCCs, where available, could provide greater scheduling flexibility. Greater perceptions of disruptions to patient care due to the intervention after the pilot may limit the long-term maintenance of the intervention. Managing dialysis care team expectations regarding how In-Tech or similar interventions may require process changes, particularly with respect to scheduling and coverage, may be critical to successful implementation.

Importantly, some of these challenges may differ substantially by clinic. We found that, although pre-pilot reach was much higher in Clinic A vs. B, with 91% vs. 20% having heard of In-Tech, Clinic A had far lower adoption of In-Tech, with participation by only one PCT in the first month of the pilot, compared to participation by up to four PCTs per month at Clinic B. Additionally, fidelity data from Clinic B, where all PCCs were attended by at least one PCT during the pilot, suggest that scheduling PCCs to accommodate at least one PCT is feasible. Although these results are limited by small sample sizes, collecting data from Clinic B on best practices around scheduling PCT shifts and PCCs may be useful for planning future studies.

The divergent adoption of In-Tech between our clinics also suggests that clinic-level contextual factors may be challenges to implementation. Such contextual factors can include organizational factors, such as the number of shifts, with more shifts allowing less between-shift time during which PCCs could potentially be scheduled; however, Clinic A and B had similar numbers of shifts. Contextual factors can also relate to team dynamics: at the end of the pilot, reports of burnout, turnover intention, and experiences of discrimination were higher, while ratings of supervisor respect and support were lower, in Clinic A than in Clinic B. Team dynamics-related factors could affect individuals’ capacity and willingness to work with other care team members to effect change. While the examination of these differences should be considered exploratory due to small sample sizes, it remains possible that they contributed to the differences in In-Tech adoption that we observed, as well as to post-implementation differences in the perceived feasibility (62% vs. 75% reporting In-Tech was implementable and 46% vs. 25% reporting it was disruptive to patient care) and appropriateness (62% vs. 79%) of In-Tech reported by participants from Clinic A vs. Clinic B. In follow-up studies, it will be essential to collect these data from target clinics and consider them in the study framework and design [9], as lower adoption of In-Tech or similar interventions is likely to diminish any effect of the intervention on outcomes of interest among dialysis care team members or patients.

The disparate perceptions of the value of In-Tech between PCTs and other dialysis care team members point to another important implementation challenge. While our small sample size precludes robust statistical testing, we found that PCTs were not only less likely than the other care team members to agree that In-Tech was valuable but also less likely to perceive that they were encouraged to share their thoughts during PCCs (60% vs. 100%) or that their input during PCCs was valued (60% vs. 100%). This gap could be addressed in multiple ways in future studies. Other care team members could be encouraged to explicitly express the value of PCT contributions during PCCs, which would encourage PCTs to continue to attend future PCCs and share important information about their patients. A guide or template for PCTs to provide feedback during PCCs, which was suggested as a potential improvement in multiple open-ended responses, could be helpful in encouraging PCT contributions. Furthermore, developing such a template with the entire dialysis care team could serve as an educational opportunity for all team members and clarify the value of the information that PCTs can provide. The inclusion of PCTs in other clinic activities relevant to patient care, such as quality improvement meetings, as suggested in some open-ended responses, might further solidify their standing as a valued member of the care team.

This study has several limitations. Due to privacy concerns, we elected not to follow survey participants longitudinally. This decision, and the potential for staff turnover during the study period, limited our ability to know whether differences in pre- vs. post-implementation responses were due to the experience of the pilot or differences in respondents between the surveys. As with any survey study, misclassification — due to volunteer or social desirability bias, or other factors such as a misunderstanding of the study purpose or specific survey items — is possible. For example, we found that more PCT respondents in Clinic A reported attending PCCs in the survey than actually attended PCCs, according to our tracking of study incentives. Further, our reliance on incentive tracking across both clinics to determine PCT participation did not allow for a detailed understanding of PCT contributions to patient cases; however, observation of the PCCs and PCT contributions was beyond the resources of our study. The offering of incentives on a per-case basis may limited our generalizability to other settings and the sustainability of the intervention beyond the pilot, although the striking difference in participation between clinics despite equal incentives suggests other contextual factors figured more prominently in PCTs’ decisions to participate. Our small sample size limited our ability to stratify estimates (e.g., by role) or to estimate associations between measures; study samples may also not have fully represented the populations of the clinics. Finally, our pilot clinics (which both had academic affiliations) may not be representative of non-academic or for-profit clinics.

## Conclusions

Despite these limitations, this pilot of a PCT-focused, dialysis care team-led intervention provides a necessary first step toward designing studies to determine the effectiveness of such interventions. These exploratory results identified important potential implementation challenges but also suggested potential solutions to inform future trials of In-Tech and similar interventions. Our results also suggest that the purpose of In-Tech — better integrating PCTs into the dialysis care team, while also obtaining novel patient information from PCTs and incorporating it into improved, patient-centered dialysis care — was generally considered valuable and important by all dialysis care team members. Future studies are warranted to demonstrate and leverage the value of PCT input in dialysis care.

## Supplementary Information

Below is the link to the electronic supplementary material.

Supplementary Material 1

Supplementary Material 2

## Data Availability

The datasets used and analysed during the current study are available from the corresponding author on reasonable request.

## Abbreviations

AIM: Acceptability of Intervention Measure
CMS: Centers for Medicare & Medicaid Services
FIM: Feasibility of Intervention Measure
HD: Hemodialysis
IAM: Intervention Appropriateness Measure
ORIC: Organizational Readiness for Implementing Change
PCC: Patient care conference
PCT: Patient care technician
PRISM: Practical, Robust Implementation and Sustainability Model
RE-AIM: Reach, Effectiveness, Adoption, Implementation and Maintenance
UCSF: University of California San Francisco
