# Supplementary material for: Inclusion of patient care technicians in hemodialysis patient care conferences: a pilot implementation study
Source: BMC Nephrol. 2026 May 30;27:452. doi: 10.1186/s12882-026-05087-6 (PMC13430903; doi:10.1186/s12882-026-05087-6)
Supplement: Supplementary file 1 — Supplementary Material 1 [file 12882_2026_5087_MOESM1_ESM.pdf]

Additional File 1: Plantinga *et al.*, Inclusion of Patient Care Technicians in Patient Care Conferences at Dialysis Clinics: A Pilot Study

Table of Contents

Supplementary Table 1. Characteristics of hemodialysis clinics participating in the In-Tech pilot 2

Supplementary Table 2. Items assessing the implementation of In-Tech in the pre- and post-intervention surveys 3

Supplementary Table 3. Survey participant characteristics and work experiences, by clinic 5

Supplementary Table 4. Perceptions and experiences of work among survey participants, by clinic 7

Supplementary Table 5. Adherence to In-Tech during pilot 9

Supplementary Figure 1. Summary of open-ended survey responses with representative quotes. 10

Supplementary Table 1. Characteristics of hemodialysis clinics participating in the In-Tech pilot

**Characteristic**[a]	**Clinic A**	**Clinic B**
Affiliation	Independent	Independent
Affiliation type	Academic	Safety-net hospital
For-profit status	Not-for-profit	Not-for-profit
No. of shifts per day	4 (MWF); 3 (TTS)	4 (MWF); 4 (TTS)
CMS five-star rating	2	3
No. of HD stations	14	13
Offers peritoneal dialysis	No	Yes
Offers home hemodialysis	No	No
Mortality rate, per 100-patient years	15.5	24.7
Hospitalization rate, per 100-patient years	131.5	160.1
Readmission rate, %	25.4%	23.4%
% prevalent patients waitlisted	46.3%	25.5%
Standardized infection ratio	0.34	1.17
% of patients with fistula	49%	66%
% of patients with long-term catheter in use	38%	21%
% of HD patients with Kt/V $\geq$1.2	93%	95%
% with calcium >10.2 mg/dl	4%	0%
% with serum phosphorus $\geq$7.0 mg/dl	21%	29%

CMS, Centers for Medicare & Medicaid Services; HD, hemodialysis; MWF, Monday/Wednesday/Friday; TTS, Tuesday/Thursday/Saturday.

[a]As of 12/31/2023.

Supplementary Table 2. Items assessing the implementation of In-Tech in the pre- and post-intervention surveys

Domain	Items: Pre-intervention	Items: Post-intervention	Responses
**Organizational readiness**	I feel confident that the organization can get people invested in implementing In-Tech.	I feel confident that the organization was able to get people invested in implementing In-Tech.	Agree<br>Somewhat agree<br>Neither agree nor disagree<br>Somewhat disagree<br>Disagree
	I will do whatever it takes to implement In-Tech.	I did whatever it took to implement In-Tech.	
	I feel confident that the people who work here can handle the challenges that might arise in implementing In-Tech.	I feel confident that the people who work here handled the challenges that arose in implementing In-Tech.	
**Feasibility**	In-Tech seems implementable in our facility.	In-Tech was implementable in our facility.	Completely agree<br>Agree<br>Neutral<br>Disagree<br>Completely disagree
	In-Tech seems easy to implement in our facility.	In-Tech was easy to implement in our facility.	
	In-Tech will be disruptive to patient care.	In-Tech was disruptive to patient care.	
**Acceptability**	I approve of In-Tech.	I approve of In-Tech.	
	I feel In-Tech is appealing.	I feel In-Tech is appealing.	
**Appropriateness**	In-Tech seems fitting for our facility.	In-Tech was fitting for our facility.	
	In-Tech seems applicable for our facility.	In-Tech was applicable for our facility.	
**Value**		Including PCTs/dialysis techs in patient care conferences was valuable to me	Completely agree<br>Agree<br>Neutral<br>Disagree<br>Completely disagree
		Including PCTs/dialysis techs in patient care conferences was valuable to the care team.	
		Including PCTs/dialysis techs in patient care conferences was valuable for patient care.	
		It was easy for me *[PCTs/dialysis techs]* to take the time to attend patient care conferences.	
		I was *[PCTs/dialysis techs were]* encouraged to share my/their thoughts during the patient care conferences that I/they attended.	
		My input *[the input of PCTs/dialysis techs]* during patient care conferences that I attended was valued by the team.	

Domain	Items		Responses
	Pre-intervention	Post-intervention	
**Value (cont.)**		Do you think it is important for PCTs/dialysis techs to attend patient care conferences? Why or why not?	Open-ended
		What are the challenges to including PCTs/dialysis techs in patient care conferences? What might make it easier? These could include individual, team, or clinic factors.	
		Do you have any thoughts on how In-Tech could be improved?	
		Anything else you want to tell us about In-Tech?	

Supplementary Table 3. Survey participant characteristics and work experiences, by clinic

Characteristic	Survey			
	Pre-intervention		Post-intervention	
	Clinic A (*N*=11)	Clinic B (*N*=10)	Clinic A (*N*= 13)	Clinic B (*N*= 24)
**Demographic**				
Age, *n* (%)				
18-34 years	3 (27.3%)	0 (0%)	4 (30.8%)	1 (4.2%)
35-49 years	5 (45.5%)	6 (60%)	6 (46.2%)	13 (54.2%)
≥50-64 years	3 (27.3%)	4 (40%)	3 (23.1%)	10 (41.7%)
Gender, *n* (%)				
Women	4 (36.4%)	8 (80%)	7 (58.3%)	16 (66.7%)
Men	7 (63.6%)	2 (20%)	5 (41.7%)	8 (33.3%)
Race[a]				
American Indian/Alaskan Native	0 (0%)	2 (22.2%)	0 (0%)	1 (4.3%)
Asian	4 (36.4%)	3 (33.3%)	5 (38.5%)	10 (43.5%)
Black	1 (9.1%)	1 (11.1%)	1 (7.7%)	2 (8.7%)
Hawaiian/Pacific Islander	3 (27.3%)	2 (22.2%)	5 (38.5%)	8 (34.8%)
White	3 (27.3%)	1 (11.1%)	2 (15.4%)	2 (8.7%)
Ethnicity, *n* (%)				
Hispanic	2 (18.2%)	0 (0%)	3 (27.3%)	1 (4.8%)
Non-Hispanic	9 (81.8%)	8 (100%)	8 (72.7%)	20 (95.2%)
Born in the United States, *n* (%)				
Yes	6 (54.5%)	5 (50.0%)	10 (76.9%)	9 (37.5%)
No	5 (45.5%)	5 (50.0%)	2 (15.4%)	15 (62.5%)
Other language spoken at home, *n* (%)				
Yes	7 (63.6%)	3 (30%)	8 (61.5%)	15 (65.2%)
No	4 (36.4%)	7 (70%)	5 (38.5%)	8 (34.8%)
**Work-related**				
Role,[a] *n* (%)				
Medical director	0 (0.0%)	0 (0.0%)	0 (0%)	1 (4.2%)
Nephrologist	0 (0.0%)	0 (0.0%)	1 (7.7%)	1 (4.2%)
Nurse manager	1 (9.1%)	1 (10.0%)	0 (0.0%)	0 (0.0%)
Charge nurse	1 (9.1%)	1 (10.0%)	1 (7.7%)	1 (4.2%)
Registered nurse	3 (27.3%)	1 (10.0%)	3 (23.1%)	5 (20.8%)
Social worker	1 (9.1%)	1 (10.0%)	1 (7.7%)	2 (8.3%)
Dietitian	1 (9.1%)	2 (20.0%)	1 (7.7%)	2 (8.3%)
Patient care technician	4 (36.4%)	2 (20.0%)	6 (46.2%)	9 (37.5%)
Other	1 (9.1%)	2 (20.0%)	3 (23.1%)	2 (8.3%)
Time in role, *n* (%)				
<1 year	1 (9.1%)	2 (20.0%)	1 (7.7%)	2 (8.4%)
1-5 years	4 (36.4%)	3 (30.0%)	5 (38.5%)	8 (33.3%)
>5 years	6 (54.5%)	5 (50.0%)	7 (53.8%)	14 (58.3%)
Working at multiple clinics, *n* (%)				
Yes	3 (27.3%)	1 (10%)	2 (15.4%)	10 (41.7%)

Characteristic	Survey			
	Pre-intervention		Post-intervention	
	Clinic A (*N*=11)	Clinic B (*N*=10)	Clinic A (*N*= 13)	Clinic B (*N*= 24)
No	8 (72.7%)	9 (90%)	11 (84.6%)	14 (58.3%)
Hours worked at clinic, *n* (%)				
≤40 hours/week	7 (63.6%)	9 (90.0%)	12 (92.3%)	16 (76.2%)
>40 hours/week	4 (36.4%)	1 (10.0%)	1 (7.7%)	5 (23.8%)
Median (IQR) patient caseload	20 (9, 56)	20 (6, 55)	34 (11, 57)	30 (12, 65)

IQR=25th-75th percentile.

[a]Totals may add to >100% since individuals could select more than one role

Supplementary Table 4. Perceptions and experiences of work among survey participants, by clinic

	Survey			
	**Pre-Intervention**		**Post-Intervention**	
**Perceptions/experiences**	**Clinic A (*N*=11)**	**Clinic B (*N*=10)**	**Clinic A (*N*=13)**	**Clinic B (*N*=24)**
Job satisfaction rating (0-10, 10=most satisfied), median (IQR)				
Overall job satisfaction	8 (5, 10)	8.5 (7, 9)	8 (7, 9)	8 (7, 10)
Burnout, *n* (%) monthly or more frequently				
Experiencing burnout	7 (63.6%)	7 (70.0%)	10 (76.9%)	8 (34.8%)
Turnover intention, *n* (%) no/not sure				
Plan to be in the same job in 1 year	3 (27.3%)	2 (20%)	4 (30.8%)	3 (12.5%)
Plan to be in the same clinic in 1 year	4 (36.4%)	2 (20.0%)	4 (30.8%)	2 (8.3%)
Extrinsic value, *n* (%) strongly agree/agree				
The pay is good.	9 (81.8%)	6 (60.0%)	12 (92.3%)	15 (62.5%)
The benefits are good	10 (90.9%)	9 (90.0%)	10 (83.3%)	20 (83.3%)
Support for continuing education is good	7 (63.6%)	8 (80.0%)	9 (69.2%)	18 (75.0%)
Intrinsic value, *n* (%) strongly agree/agree				
My patients give me a reason to come to work every day	10 (90.9%)	8 (80.0%)	11(84.6%)	20 (83.3%)
Expectations and mobility, *n* (%) strongly agree/agree				
Job security is good	8 (72.7%)	8 (80.0%)	9 (69.2%)	20 (83.3%)
Chances for promotion are good.	4 (36.4%)	5 (50.0%)	7 (53.8%)	14 (58.3%)
Promotions are handled fairly.	4 (36.4%)	4 (40.0%)	7 (53.8%)	16 (66.7%)
My job measures up to the sort of job I wanted when I took it.	7 (63.6%)	9 (90.0%)	9 (69.2%)	19 (79.2%)
Respect, *n* (%) strongly agree/agree				
My supervisor treats me as an equal member of the healthcare team	8 (72.7%)	8 (80.0%)	8 (61.5%)	22 (91.7%)
My supervisor listens carefully to my observations and opinions	7 (63.6%)	10 (100%)	7 (53.8%)	21 (91.3%)
My other coworkers treat me as an equal member of the healthcare	10 (90.9%)	9 (90.0%)	12 (92.3%)	20 (87%)
My other coworkers listen carefully to my observations and opinions	11 (100%)	9 (90.0%)	11 (84.6%)	21 (87.5%)
Autonomy, *n* (%) strongly agree/agree				
It is basically my own responsibility to decide how my job gets done	7 (63.6%)	10 (100%)	7 (53.8%)	22 (91.7%)
I have input into patient care planning	6 (54.5%)	10 (100%)	9 (69.2%)	20 (83.3%)
Discrimination/violence, *n* (%) rarely/sometimes/often/always				
I have been treated unfairly at work because of my race, ethnic group, gender, age, disability status, or other personal characteristic	8 (72.7%)	2 (20.0%)	10 (76.9%)	6 (25.0%)

Perceptions/experiences	Survey			
	Pre-Intervention		Post-Intervention	
	**Clinic A (*N*=11)**	**Clinic B (*N*=10)**	**Clinic A (*N*=13)**	**Clinic B (*N*=24)**
I have experienced threats of violence or physical abuse, or actual abuse, at work	5 (45.5%)	3 (30.0%)	4 (30.8%)	7 (29.2%)
Perceptions of patient care, *n* (%) strongly agree/agree				
Sometimes our staff take out their bad days on the patients	0 (0.0%)	1 (10.0%)	2 (15.4%)	1 (4.2%)
Some staff are hostile toward patients	4 (40.0%)	2 (20.0%)	4 (33.3%)	1 (4.3%)
I treat patients like I would like to be treated	10 (90.9%)	10 (100%)	11 (84.6%)	22 (95.7%)
Patient areas are kept clean here	10 (90.9%)	9 (90.0%)	10 (76.9%)	19 (79.2%)
All personnel take responsibility for answering patient alarms	8 (72.7%)	9 (90.0%)	7 (53.8%)	20 (83.3%)

IQR, interquartile range (25th-75th percentile).

Supplementary Table 5. Adherence to In-Tech during pilot

	n/N (%)					
	**PCTs attending at least one PCC**		**PCCs attended by at least one PCT**		**Patient cases discussed by a PCT**	
**Month:**	**Clinic A**	**Clinic B**	**Clinic A**	**Clinic B**	**Clinic A**	**Clinic B**
1	1/14 (7.1%)	2/14 (14.3%)	—*	4/4 (100%)	—*	7/12 (58.3%)
2	0/14 (0.0%)	4/14 (28.6%)	0.0%*	4/4 (100%)	0.0%*	14/14 (100%)
3	0/14 (0.0%)	4/14 (28.6%)	0.0%*	5/5 (100%)	0.0%*	9/10 (90.0%)
4	0/14 (0.0%)	4/14 (28.6%)	0.0%*	5/5 (100%)	0.0%*	12/19 (63.2%)
5	0/14 (0.0%)	4/14 (28.6%)	0.0%*	5/5 (100%)	0.0%*	12/14 (85.7%)
6	0/14 (0.0%)	4/13 (30.8%)	0.0%*	5/5 (100%)	0.0%*	13/13 (100%)

*Denominator information not provided by clinic. PCC, patient care conference; PCT, patient care technician.

Supplementary Figure 1. Summary of open-ended survey responses with representative quotes.

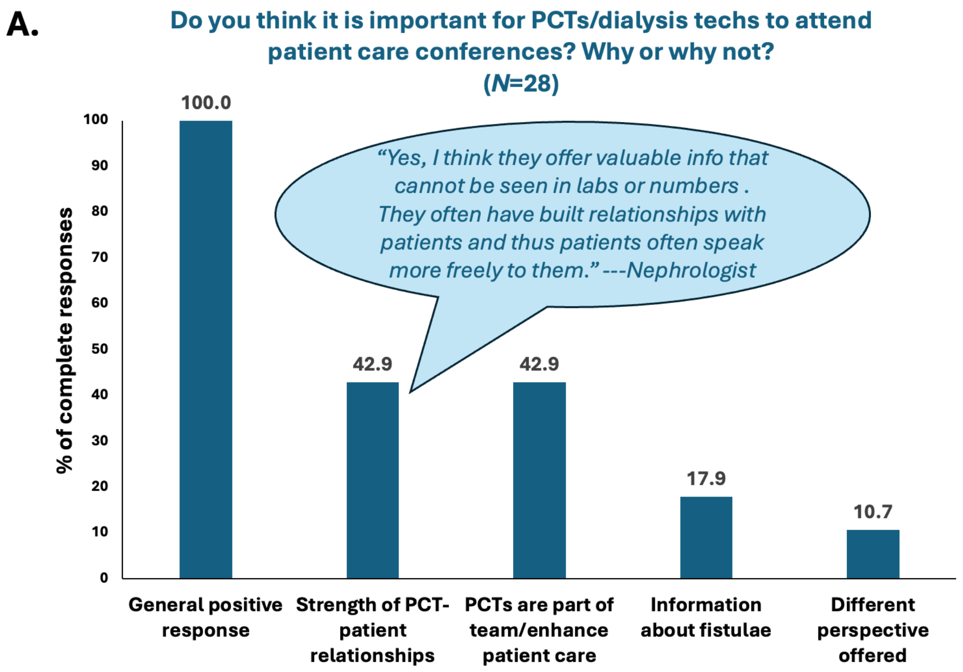

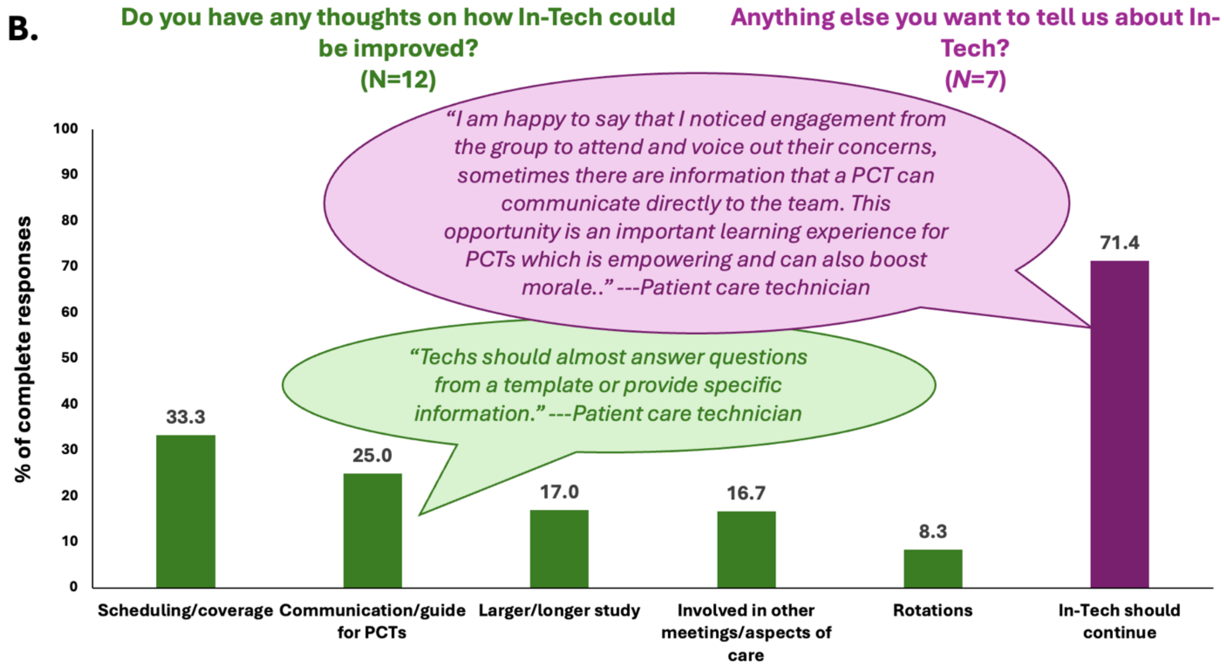

C.

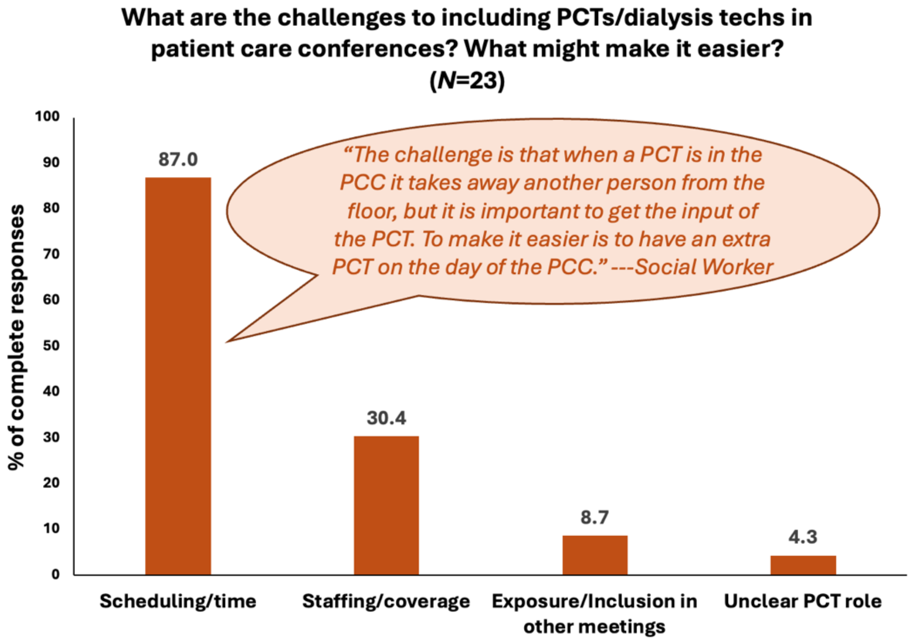
What are the challenges to including PCTs/dialysis techs in patient care conferences? What might make it easier? (N=23)
% of complete responses
100
90
80
70
60
50
40
30
20
10
0
87.0
30.4
8.7
4.3
"The challenge is that when a PCT is in the PCC it takes away another person from the floor, but it is important to get the input of the PCT. To make it easier is to have an extra PCT on the day of the PCC." ---Social Worker
Scheduling/time
Staffing/coverage
Exposure/Inclusion in other meetings
Unclear PCT role
