# Supplementary material for: Inclusion of patient care technicians in hemodialysis patient care conferences: a pilot implementation study
Source: BMC Nephrol. 2026 May 30;27:452. doi: 10.1186/s12882-026-05087-6 (PMC13430903; doi:10.1186/s12882-026-05087-6)
Supplement: Supplementary file 2 — Supplementary Material 2 [file 12882_2026_5087_MOESM2_ESM.pdf]

Additional File 2: Plantinga *et al.*, Inclusion of Patient Care Technicians in Patient Care Conferences at Dialysis Clinics: A Pilot Study

Table of Contents

In-Tech Pre-Intervention Survey ***2***

In-Tech Post-Intervention Survey ***9***

In-Tech Pre-Intervention Survey

Item/text	Responses	Source
Researchers at UCSF are conducting a pilot study to understand the effect of the participation of dialysis patient care technicians (PCTs or "techs") in monthly interdisciplinary patient care conferences or rounds on team dynamics and on the quality of patient care.	---	*Ad hoc*
Are you currently providing care to patients receiving dialysis at your facility in one of the following roles?	1, Medical director | 2, Nephrologist | 3, Advanced practice provider (NP or PA) | 4, Nurse manager | 5, Charge nurse | 6, Registered nurse | 7, Social worker | 8, Dietitian | 9, Patient care technician | 10, Biomed technician | 11, Other role	
<consent form>		*University of California San Francisco Institutional Review Board template*
If you wish to take the survey, please click "I consent to participate" to proceed.	1, I consent to participate | 2, I do not consent to participate	*Ad hoc*
How long have you been a <role>?	1, < 6 months | 2, 6-12 months | 3, >1-5 years | 4, >5 years	
How long have you been a <role> at this facility?	1, < 6 months | 2, 6-12 months | 3, >1-5 years | 4, >5 years	
Are you a temporary employee at this facility?	1, Yes | 2, No	
Do you currently work at another dialysis facility?	1, Yes | 2, No	
On average, how many hours per week do you work at this facility?	1, 20 hours or less | 2, 21-40 hours | 3, 41-60 hours | 4, 61+ hours	

Item/text	Responses	Source
On average, how many in-center hemodialysis shifts do you cover per week at *this* facility?	Integer	*Ad hoc*
Thinking about your primary job at this facility, about how many in-center hemodialysis patients in total do you care for in a month at your facility? "Care" refers to any health care, service or support provided in the dialysis setting. If you work multiple shifts or provide multiple treatments per patients, please provide the number of patients (for example, if you generally work 4 shifts per week with 5 patients each over the course of a month, you care for 20 patients total in a month).	Integer	
On a scale of 0 to 10, with 0 being very dissatisfied and 10 being completely satisfied, how satisfied are you with your current job?<br><br>Please move the slider to the position/number you would like.	1=Completely dissatisfied | 5=Neither satisfied nor dissatisfied | 10=Completely satisfied	Dill JS, Morgan JC, Marshall VW, Pruchno R. Contingency, employment intentions, and retention of vulnerable low-wage workers: an examination of nursing assistants in nursing homes. Gerontologist. 2013 Apr;53(2):222-34.<br>*(No modifications)*
The pay is good.	1, Strongly agree | 2, Agree | 3, Neither agree nor disagree | 4, Disagree | 5, Strongly Disagree	
The benefits (e.g., health insurance, leave, retirement) are good.	1, Strongly agree | 2, Agree | 3, Neither agree nor disagree | 4, Disagree | 5, Strongly Disagree	

Item/text	Responses	Source
Support for continuing education is good.	1, Strongly agree | 2, Agree | 3, Neither agree nor disagree | 4, Disagree | 5, Strongly Disagree	Dill JS, Morgan JC, Marshall VW, Pruchno R. Contingency, employment intentions, and retention of vulnerable low-wage workers: an examination of nursing assistants in nursing homes. Gerontologist. 2013 Apr;53(2):222-34. *(No modifications)*
The job security is good.	1, Strongly agree | 2, Agree | 3, Neither agree nor disagree | 4, Disagree | 5, Strongly Disagree	
The chances for promotion are good.	1, Strongly agree | 2, Agree | 3, Neither agree nor disagree | 4, Disagree | 5, Strongly Disagree	
Promotions are handled fairly.	1, Strongly agree | 2, Agree | 3, Neither agree nor disagree | 4, Disagree | 5, Strongly Disagree	
My job measures up to the sort of job I wanted when I took it.	1, Strongly agree | 2, Agree | 3, Neither agree nor disagree | 4, Disagree | 5, Strongly Disagree	
Job burnout is defined as a chronic state of physical and emotional depletion that results from excessive work demands.		West CP, Dyrbye LN, Sloan JA, Shanafelt TD. Single item measures of emotional exhaustion and depersonalization are useful for assessing burnout in medical professionals. J Gen Intern Med. 2009 Dec;24(12):1318-21 *(No modifications)*
Please tell us how frequently you feel burned out from your work.	1, Never | 2, A few times a year or less | 3, Once a month or less | 4, A few times a month | 5, Once a week | 6, A few times a week | 7, Every day	
Do you plan to be working in the same job in a year?	1, Yes | 2, No | 3, Not sure	Dill JS, Morgan JC, Marshall VW, Pruchno R. Contingency, employment intentions, and retention of vulnerable low-wage workers: an examination of nursing assistants in nursing homes. Gerontologist. 2013 Apr;53(2):222-34 *(Modification: changed 3 years to 1 year)*
Do you plan to be working in the same facility in a year?	1, Yes | 2, No | 3, Not sure	
My supervisor treats me as an equal member of the healthcare team.	1, Strongly agree | 2, Agree | 3, Neither agree nor disagree | 4, Disagree | 5, Strongly Disagree	

Item/text	Responses	Source
My supervisor listens carefully to my observations and opinions.	1, Strongly agree | 2, Agree | 3, Neither agree nor disagree | 4, Disagree | 5, Strongly Disagree	Dill JS, Morgan JC, Marshall VW, Pruchno R. Contingency, employment intentions, and retention of vulnerable low-wage workers: an examination of nursing assistants in nursing homes. Gerontologist. 2013 Apr;53(2):222-34 *(No modifications)*
My other coworkers treat me as an equal member of the healthcare team.	1, Strongly agree | 2, Agree | 3, Neither agree nor disagree | 4, Disagree | 5, Strongly Disagree	
My other coworkers listen carefully to my observations and opinions.	1, Strongly agree | 2, Agree | 3, Neither agree nor disagree | 4, Disagree | 5, Strongly Disagree	
It is basically my own responsibility to decide how my job gets done.	1, Strongly agree | 2, Agree | 3, Neither agree nor disagree | 4, Disagree | 5, Strongly Disagree	
I have input into patient care planning.	1, Strongly agree | 2, Agree | 3, Neither agree nor disagree | 4, Disagree | 5, Strongly Disagree	
I have been treated unfairly at work because of my race, ethnic group, gender, age, disability status, or other personal characteristic.	1, Never | 2, Rarely | 3, Sometimes | 4, Often | 5, Almost all of the time	Negative Acts Questionnaire-Revised: Einarsen S, Hoel H, Notelaers G. Measuring exposure to bullying and harassment at work: Validity, factor structure and psychometric properties of the Negative Acts Questionnaire-Revised. Work Stress. 2009;23(1): 24-44 *(Modifications: included only two items; sexual harassment item modified to include all discrimination)*
I have experienced threats of violence or physical abuse, or actual abuse, at work.	1, Never | 2, Rarely | 3, Sometimes | 4, Often | 5, Almost all of the time	
Sometimes our staff take out their bad days on the patients.	1, Strongly agree | 2, Agree | 3, Neither agree nor disagree | 4, Disagree | 5, Strongly Disagree	
Some staff are hostile toward patients.	1, Strongly agree | 2, Agree | 3, Neither agree nor disagree | 4, Disagree | 5, Strongly Disagree	

Item/text	Responses	Source
I treat patients like I would like to be treated.	1, Strongly agree | 2, Agree | 3, Neither agree nor disagree | 4, Disagree | 5, Strongly Disagree	Dill JS, Morgan JC, Marshall VW, Pruchno R. Contingency, employment intentions, and retention of vulnerable low-wage workers: an examination of nursing assistants in nursing homes. Gerontologist. 2013 Apr;53(2):222-34. *(Modification: replaced "call lights" replaced with "patient alarms")*
Patient areas are kept clean here.	1, Strongly agree | 2, Agree | 3, Neither agree nor disagree | 4, Disagree | 5, Strongly Disagree	
All personnel take responsibility for answering patient alarms.	1, Strongly agree | 2, Agree | 3, Neither agree nor disagree | 4, Disagree | 5, Strongly Disagree	
My patients give me a reason to come to work every day.	1, Strongly agree | 2, Agree | 3, Neither agree nor disagree | 4, Disagree | 5, Strongly Disagree	
Have you heard about In-Tech? (check all that apply)	1, Yes - from study team presentations | 2, Yes - from coworkers | 3, No | 4, Other	*Ad hoc*
I feel confident that the organization can get people invested in implementing In-Tech.	1, Agree | 2, Somewhat agree | 3, Neither agree nor disagree | 4, Somewhat disagree | 5, Disagree	Organizational Readiness for Implementing Change (ORIC) Shea CM, Jacobs SR, Esserman DA, Bruce K, Weiner BJ. Organizational readiness for implementing change: a psychometric assessment of a new measure. Implement Sci. 2014;9:7 *(Modifications: used only three items, changed "people who work here" to "I")*
I will do whatever it takes to implement In-Tech.	1, Agree | 2, Somewhat agree | 3, Neither agree nor disagree | 4, Somewhat disagree | 5, Disagree	
I feel confident that the people who work here can handle the challenges that might arise in implementing In-Tech.	1, Agree | 2, Somewhat agree | 3, Neither agree nor disagree | 4, Somewhat disagree | 5, Disagree	
I approve of In-Tech.	1, Completely agree | 2, Agree | 3, Neutral | 4, Disagree | 5, Completely disagree	Acceptability of Intervention Measure (AIM) Weiner BJ, Lewis CC, Stanick C, Powell BJ, Dorsey CN, Clary AS, Boynton MH, Halko H. Psychometric assessment of three newly developed implementation outcome measures. Implement Sci. 2017;12(1):108
I feel In-Tech is appealing.	1, Completely agree | 2, Agree | 3, Neutral | 4, Disagree | 5, Completely disagree	

Item/text	Responses	Source
		(Modifications: used only two items, included "In-Tech," changed "meets my approval" to "I approve of")
In-Tech seems fitting for our facility.	1, Completely agree | 2, Agree | 3, Neutral | 4, Disagree | 5, Completely disagree	Intervention Appropriateness Measure (IAM)<br>Weiner BJ, Lewis CC, Stanick C, Powell BJ, Dorsey CN, Clary AS, Boynton MH, Halko H. Psychometric assessment of three newly developed implementation outcome measures. Implement Sci. 2017;12(1):108<br>*(Modifications: used only two items, included "In-Tech," added "for our facility")*
In-Tech seems applicable for our facility.	1, Completely agree | 2, Agree | 3, Neutral | 4, Disagree | 5, Completely disagree	
In-Tech seems implementable in our facilty.	1, Completely agree | 2, Agree | 3, Neutral | 4, Disagree | 5, Completely disagree	Feasibility of Intervention Measure (FIM)<br>Weiner BJ, Lewis CC, Stanick C, Powell BJ, Dorsey CN, Clary AS, Boynton MH, Halko H. Psychometric assessment of three newly developed implementation outcome measures. Implement Sci. 2017;12(1):108<br>*(Modifications: used only two items, included "In-Tech," changed "easy to use" to "easy to implement," added "in our facility")*
In-Tech seems easy to implement in our facility.	1, Completely agree | 2, Agree | 3, Neutral | 4, Disagree | 5, Completely disagree	
In-Tech will be disruptive to patient care.	1, Completely agree | 2, Agree | 3, Neutral | 4, Disagree | 5, Completely disagree	
Do you have any other thoughts about In-Tech that you would like to share? (for example, whether you like the idea, how you expect it to work, etc.)	Open-ended	*Ad hoc*
What is your age?	1, 18-34 years | 2, 35-49 years | 3, 50-64 years | 4, 65+ years | 9, Prefer not to answer	\

Item/text	Responses	Source
What is your gender? (check all that apply)	1, Female | 2, Male | 3, Nonbinary | 4, Other | 5, Prefer not to answer	*Ad hoc*
Are you of Hispanic, Latino, or Spanish origin? (check all that apply)	1, No, not of Hispanic, Latino, or Spanish origin | 2, Yes, Mexican, Mexican-American, Chicano | 3, Yes, Puerto Rican | 4, Yes, Cuban | 5, Yes, another Hispanic, Latino, or Spanish origin (e.g., Salvadoran, Guatemalan, Dominican, Colombian, Spaniard, Ecuadorian, etc.) | 9, Prefer not to answer	U.S. Census 2020 *(No modifications)*
What is your race? (check all that apply)	1, White | 2, Black or African-American | 3, American Indian/Alaskan Native | 4, Asian [includes Chinese, Vietnamese, Korean, Japanese, Asian Indian, and Other Asian (e.g., Pakistani, Cambodian, Hmong)] | 5, Hawaiian or Pacific Islander [includes Native Hawaiian, Filipino, Samoan, Chamorro, and Other Pacific Islander (e.g., Tongan, Fijian)] | 6, Some other race | 99, Prefer not to answer	
Where were you born?	1, United States | 2, Outside of the United States | 9, Prefer not to answer	*Ad hoc*
Do you speak a language other than English at home?	1, Yes | 2, No | 9, Prefer not to answer	

In-Tech Post-Intervention Survey

Item/text	Responses	Source
Are you currently providing care to patients receiving dialysis at your facility in one of the following roles?(check all that apply)	1, Medical director | 2, Nephrologist | 3, Advanced practice provider (NP or PA) | 4, Nurse manager | 5, Charge nurse | 6, Registered nurse | 7, Social worker | 8, Dietitian | 9, Patient care technician | 10, Biomed technician | 11, Other role	*Ad hoc*
Great! You are eligible to participate. Please continue to the survey consent.		
<consent form>		*University of California San Francisco Institutional Review Board template*
If you wish to take the survey, please click "I consent to participate" to proceed.	1, I consent to participate	*Ad hoc*
How long have you been a <role>?	1, < 6 months | 2, 6-12 months | 3, >1-5 years | 4, >5 years	
How long have you been a <role> at this facility	1, < 6 months | 2, 6-12 months | 3, >1-5 years | 4, >5 years	
Are you a temporary employee at your facility?	1, Yes | 2, No	
Do you currently work at another dialysis facility?	1, Yes | 2, No	
On average, how many hours per week do you work at your facility?	1, 20 hours or less | 2, 21-40 hours | 3, 41-60 hours | 4, 61+ hours	
On average, how many in-center hemodialysis shifts do you cover per week at your facility?	Integer	
On average, about how many patients are you responsible for during a single shift?	Integer	
About how many in-center hemodialysis patients in total are	Integer	

Item/text	Responses	Source
you currently responsible for at your facility? *<non-shift workers>*		
Have you heard about In-Tech? (check all that apply)	1, Yes - from study team presentations | 2, Yes - from coworkers | 3, No | 4, Other	*Ad hoc*
I participated in at least one patient care conference over the last 6 months.	1, Yes | 2, No | 3, I don't know	
I participated in at least one patient care conference in the last 6 months where a PCT/technician was present.	1, Yes | 2, No | 3, I don't know	
Including PCTs/dialysis techs in patient care conferences was valuable to me.	1, Completely agree | 2, Agree | 3, Neutral | 4, Disagree | 5, Completely disagree	
Including PCTs/dialysis techs in patient care conferences was valuable to the care team.	1, Completely agree | 2, Agree | 3, Neutral | 4, Disagree | 5, Completely disagree	
Including PCTs/dialysis techs in patient care conferences was valuable for patient care.	1, Completely agree | 2, Agree | 3, Neutral | 4, Disagree | 5, Completely disagree	
It was easy for me to take the time to attend patient care conferences.	1, Completely agree | 2, Agree | 3, Neutral | 4, Disagree | 5, Completely disagree	
I was encouraged to share my thoughts during the patient care conferences that I attended.	1, Completely agree | 2, Agree | 3, Neutral | 4, Disagree | 5, Completely disagree	
My input during patient care conferences that I attended was valued by the team.	1, Completely agree | 2, Agree | 3, Neutral | 4, Disagree | 5, Completely disagree	
It was easy for the PCTs/dialysis techs to take the time to attend patient care conferences.	1, Completely agree | 2, Agree | 3, Neutral | 4, Disagree | 5, Completely disagree	

<table>
<tr><th>Item/text</th><th>Responses</th><th>Source</th></tr>
<tr><td>When PCTs/dialysis techs attended patient care conferences, they were encouraged to share their thoughts.</td><td>1, Completely agree | 2, Agree | 3, Neutral | 4, Disagree | 5, Completely disagree</td><td rowspan="6">Ad hoc</td></tr>
<tr><td>The PCTs'/dialysis techs' input during patient care conferences was valued by the team.</td><td>1, Completely agree | 2, Agree | 3, Neutral | 4, Disagree | 5, Completely disagree</td></tr>
<tr><td>Do you think it is important for PCTs/dialysis techs to attend patient care conferences? Why or why not?</td><td>Open-ended</td></tr>
<tr><td>What are the challenges to including PCTs/dialysis techs in patient care conferences? What might make it easier? These could include individual, team, or clinic factors.</td><td>Open-ended</td></tr>
<tr><td>Do you have any thoughts on how In-Tech could be improved?</td><td>Open-ended</td></tr>
<tr><td>Anything else you'd like to add about In-Tech?</td><td>Open-ended</td></tr>
<tr><td>I feel confident that the organization was able to get people invested in implementing In-Tech.</td><td>1, Agree | 2, Somewhat agree | 3, Neither agree nor disagree | 4, Somewhat disagree | 5, Disagree</td><td rowspan="3">Organizational Readiness for Implementing Change (ORIC)
Shea CM, Jacobs SR, Esserman DA, Bruce K, Weiner BJ. Organizational readiness for implementing change: a psychometric assessment of a new measure. Implement Sci. 2014;9:7
(Modifications: used only three items, changed “people who work here” to “I,” changed to past tense)</td></tr>
<tr><td>I did whatever it took to implement In-Tech.</td><td>1, Agree | 2, Somewhat agree | 3, Neither agree nor disagree | 4, Somewhat disagree | 5, Disagree</td></tr>
<tr><td>I feel confident that the people who work here handled the challenges that arose in implementing In-Tech.</td><td>1, Agree | 2, Somewhat agree | 3, Neither agree nor disagree | 4, Somewhat disagree | 5, Disagree</td></tr>
</table>

Item/text	Responses	Source
I approve of In-Tech.	1, Completely agree | 2, Agree | 3, Neutral | 4, Disagree | 5, Completely disagree	Acceptability of Intervention Measure (AIM) Weiner BJ, Lewis CC, Stanick C, Powell BJ, Dorsey CN, Clary AS, Boynton MH, Halko H. Psychometric assessment of three newly developed implementation outcome measures. Implement Sci. 2017;12(1):108 *(Modifications: used only two items, included "In-Tech," changed "meets my approval" to "I approve of")*
I feel In-Tech is appealing.	1, Completely agree | 2, Agree | 3, Neutral | 4, Disagree | 5, Completely disagree	
In-Tech was fitting for our facility.	1, Completely agree | 2, Agree | 3, Neutral | 4, Disagree | 5, Completely disagree	Intervention Appropriateness Measure (IAM) Weiner BJ, Lewis CC, Stanick C, Powell BJ, Dorsey CN, Clary AS, Boynton MH, Halko H. Psychometric assessment of three newly developed implementation outcome measures. Implement Sci. 2017;12(1):108 *(Modifications: used only two items, included "In-Tech," added "for our facility")*
In-Tech was applicable for our facility.	1, Completely agree | 2, Agree | 3, Neutral | 4, Disagree | 5, Completely disagree	
In-Tech was implementable in our facilty.	1, Completely agree | 2, Agree | 3, Neutral | 4, Disagree | 5, Completely disagree	Feasibility of Intervention Measure (FIM) Weiner BJ, Lewis CC, Stanick C, Powell BJ, Dorsey CN, Clary AS, Boynton MH, Halko H. Psychometric assessment of three newly developed implementation outcome measures. Implement Sci. 2017;12(1):108 *(Modifications: used only two items, included "In-Tech," changed "easy to use" to "easy to implement," added "in our facility")*
In-Tech was easy to implement in our facility.	1, Completely agree | 2, Agree | 3, Neutral | 4, Disagree | 5, Completely disagree	
In-Tech was disruptive to patient care.	1, Completely agree | 2, Agree | 3, Neutral | 4, Disagree | 5, Completely disagree	*Ad hoc*

Item/text	Responses	Source
On a scale of 0 to 10, with 0 being very dissatisfied and 10 being completely satisfied, how satisfied are you with your current job?<br><br>Please move the slider to the position/number you would like.	1=Completely dissatisfied | 5=Neither satisfied nor dissatisfied | 10=Completely satisfied	Dill JS, Morgan JC, Marshall VW, Pruchno R. Contingency, employment intentions, and retention of vulnerable low-wage workers: an examination of nursing assistants in nursing homes. Gerontologist. 2013 Apr;53(2):222-34. *(No modifications)*
The pay is good.	1, Strongly agree | 2, Agree | 3, Neither agree nor disagree | 4, Disagree | 5, Strongly Disagree	
The benefits (e.g., health insurance, leave, retirement) are good.	1, Strongly agree | 2, Agree | 3, Neither agree nor disagree | 4, Disagree | 5, Strongly Disagree	
Support for continuing education is good.	1, Strongly agree | 2, Agree | 3, Neither agree nor disagree | 4, Disagree | 5, Strongly Disagree	
The job security is good.	1, Strongly agree | 2, Agree | 3, Neither agree nor disagree | 4, Disagree | 5, Strongly Disagree	
The chances for promotion are good.	1, Strongly agree | 2, Agree | 3, Neither agree nor disagree | 4, Disagree | 5, Strongly Disagree	
Promotions are handled fairly.	1, Strongly agree | 2, Agree | 3, Neither agree nor disagree | 4, Disagree | 5, Strongly Disagree	
My job measures up to the sort of job I wanted when I took it.	1, Strongly agree | 2, Agree | 3, Neither agree nor disagree | 4, Disagree | 5, Strongly Disagree	
Job burnout is defined as a chronic state of physical and emotional depletion that results from excessive work demands.		West CP, Dyrbye LN, Sloan JA, Shanafelt TD. Single item measures of emotional

Item/text	Responses	Source
Please tell us how frequently you feel burned out from your work.	1, Never | 2, A few times a year or less | 3, Once a month or less | 4, A few times a month | 5, Once a week | 6, A few times a week | 7, Every day	exhaustion and depersonalization are useful for assessing burnout in medical professionals. J Gen Intern Med. 2009 Dec;24(12):1318-21 *(No modifications)*
Do you plan to be working in the same job in a year?	1, Yes | 2, No | 3, Not sure	Dill JS, Morgan JC, Marshall VW, Pruchno R. Contingency, employment intentions, and retention of vulnerable low-wage workers: an examination of nursing assistants in nursing homes. Gerontologist. 2013 Apr;53(2):222-34 *(Modification: changed 3 years to 1 year)*
you plan to be working in the same facility in a year?	1, Yes | 2, No | 3, Not sure	
My supervisor treats me as an equal member of the healthcare team.	1, Strongly agree | 2, Agree | 3, Neither agree nor disagree | 4, Disagree | 5, Strongly Disagree	Dill JS, Morgan JC, Marshall VW, Pruchno R. Contingency, employment intentions, and retention of vulnerable low-wage workers: an examination of nursing assistants in nursing homes. Gerontologist. 2013 Apr;53(2):222-34. *(No modifications)*
My supervisor listens carefully to my observations and opinions.	1, Strongly agree | 2, Agree | 3, Neither agree nor disagree | 4, Disagree | 5, Strongly Disagree	
My other coworkers treat me as an equal member of the healthcare team.	1, Strongly agree | 2, Agree | 3, Neither agree nor disagree | 4, Disagree | 5, Strongly Disagree	
My other coworkers listen carefully to my observations and opinions.	1, Strongly agree | 2, Agree | 3, Neither agree nor disagree | 4, Disagree | 5, Strongly Disagree	
It is basically my own responsibility to decide how my job gets done.	1, Strongly agree | 2, Agree | 3, Neither agree nor disagree | 4, Disagree | 5, Strongly Disagree	
I have input into patient care planning.	1, Strongly agree | 2, Agree | 3, Neither agree nor disagree | 4, Disagree | 5, Strongly Disagree	

Item/text	Responses	Source
I have been treated unfairly at work because of my race, ethnic group, gender, age, disability status, or other personal characteristic.	1, Never | 2, Rarely | 3, Sometimes | 4, Often | 5, Almost all of the time	Negative Acts Questionnaire-Revised: Einarsen S, Hoel H, Notelaers G. Measuring exposure to bullying and harassment at work: Validity, factor structure and psychometric properties of the Negative Acts Questionnaire-Revised. Work Stress. 2009;23(1): 24-44 *(Modifications: included only two items; sexual harassment item modified to include all discrimination)*
I have experienced threats of violence or physical abuse, or actual abuse, at work.	1, Never | 2, Rarely | 3, Sometimes | 4, Often | 5, Almost all of the time	
Sometimes our staff take out their bad days on the patients.	1, Strongly agree | 2, Agree | 3, Neither agree nor disagree | 4, Disagree | 5, Strongly Disagree	Dill JS, Morgan JC, Marshall VW, Pruchno R. Contingency, employment intentions, and retention of vulnerable low-wage workers: an examination of nursing assistants in nursing homes. Gerontologist. 2013 Apr;53(2):222-34. *(Modification: replaced "call lights" replaced with "patient alarms")*
Some staff are hostile toward patients.	1, Strongly agree | 2, Agree | 3, Neither agree nor disagree | 4, Disagree | 5, Strongly Disagree	
I treat patients like I would like to be treated.	1, Strongly agree | 2, Agree | 3, Neither agree nor disagree | 4, Disagree | 5, Strongly Disagree	
Patient areas are kept clean here.	1, Strongly agree | 2, Agree | 3, Neither agree nor disagree | 4, Disagree | 5, Strongly Disagree	
All personnel take responsibility for answering patient alarms.	1, Strongly agree | 2, Agree | 3, Neither agree nor disagree | 4, Disagree | 5, Strongly Disagree	
My patients give me a reason to come to work every day.	1, Strongly agree | 2, Agree | 3, Neither agree nor disagree | 4, Disagree | 5, Strongly Disagree	
What is your age?	1, 18-34 years | 2, 35-49 years | 3, 50-64 years | 4, 65+ years | 9, Prefer not to answer	*Ad hoc*
What is your gender? (check all that apply)	1, Female | 2, Male | 3, Nonbinary | 4, Other | 5, Prefer not to answer	

Item/text	Responses	Source
Are you of Hispanic, Latino, or Spanish origin? (check all that apply)	1, No, not of Hispanic, Latino, or Spanish origin | 2, Yes, Mexican, Mexican-American, Chicano | 3, Yes, Puerto Rican | 4, Yes, Cuban | 5, Yes, another Hispanic, Latino, or Spanish origin (e.g., Salvadoran, Guatemalan, Dominican, Colombian, Spaniard, Ecuadorian, etc.) | 9, Prefer not to answer	U.S. Census 2020 *(No modifications)*
What is your race? (check all that apply)>	1, White | 2, Black or African-American | 3, American Indian/Alaskan Native | 4, Asian [includes Chinese, Vietnamese, Korean, Japanese, Asian Indian, and Other Asian (e.g., Pakistani, Cambodian, Hmong)] | 5, Hawaiian or Pacific Islander [includes Native Hawaiian, Filipino, Samoan, Chamorro, and Other Pacific Islander (e.g., Tongan, Fijian)] | 6, Some other race | 99, Prefer not to answer	
Where were you born?	1, United States | 2, Outside of the United States | 9, Prefer not to answer	*Ad hoc*
Do you speak a language other than English at home?	1, Yes | 2, No | 9, Prefer not to answer	
